# GIANT CELL TUMOR OF BONE: A MULTICENTER EPIDEMIOLOGICAL STUDY IN BRAZIL

**DOI:** 10.1590/1413-785220243201e273066

**Published:** 2024-03-22

**Authors:** Ricardo Gehrke Becker, Carlos Roberto Galia, Julie Francine Cerutti Santos Pestilho, Bruno Pereira Antunes, André Mathias Baptista, Alex Guedes

**Affiliations:** 1 Hospital de Clínicas de Porto Alegre (HCPA), Department of Orthopedics and Trauma, Porto Alegre, RS, Brazil.; 2 Instituto do Câncer Infantil, Porto Alegre, RS, Brazil.; 3 Universidade de São Paulo (USP), School of Medicine, São Paulo, SP, Brazil.; 4 Santa Casa de Misericórdia da Bahia, Hospital Santa Izabel, Orthopedic Oncology Group, Salvador, BA, Brazil.

**Keywords:** Bone Neoplasms, Giant Cell Tumor of Bone, Curettage, Recurrence, Neoplasias Ósseas, Tumor de Células Gigantes do Osso, Curetagem, Recidiva

## Abstract

**Introduction:**

Giant cell tumor of bone (GCTB) mainly affects young adults’ long bone epiphyses, threatening bone strength and joint function. Surgery is the primary treatment, although post-surgery recurrence is significant. This study analyzes patient profiles, treatments, and outcomes for GCTB in Brazil.

**Methods:**

We retrospectively assessed local recurrence, metastasis, and treatment approaches in 643 GCTB patients across 16 Brazilian centers (1989-2021), considering regional differences.

**Results:**

5.1% (n=33) developed pulmonary metastases, 14.3% (n=92) had pathological fractures, and the local recurrence rate was 18.2% (n=114). Higher rates of pulmonary metastases (12.1%) and advanced tumors (Campanacci III, 88.9%) were noted in lower-income North and Northeast regions. The North also had more pathological fractures (33.3%), extensive resections (61.1%), and amputations (27.8%). These regions faced longer surgical delays (36-39 days) than the South and Southeast (27-33 days).

**Conclusions:**

Our findings corroborate international data, underscoring regional disparities in Brazil that may lead to worse outcomes in disadvantaged areas. This highlights the need for improved orthopedic oncology care in Brazil’s economically and structurally challenged regions. ***Level of Evidence III; Retrospective Cohort.***

## RESUMO

**Introdução:**

O tumor de células gigantes do osso (TCG) atinge principalmente epífises de ossos longos em adultos jovens, impactando a resistência óssea e a funcionalidade articular. O tratamento principal é cirúrgico, mas há significativa recorrência pós-operatória. Este estudo analisa o perfil de pacientes e tumores de TCG no Brasil, abordagens de tratamento e resultados.

**Métodos:**

Avaliamos retrospectivamente taxas de recorrência, metástase e tratamentos em 643 pacientes tratados em 16 centros brasileiros de 1989 a 2021, considerando a distribuição geopolítica.

**Resultados:**

5,1% desenvolveram metástases pulmonares e 14,3% tiveram fraturas patológicas. A recorrência local foi de 18,2%. Regiões economicamente menos favorecidas, como Norte e Nordeste, mostraram maiores incidências de metástases pulmonares (12,1%) e tumores avançados (Campanacci III, 88,9%). O Norte teve alta ocorrência de fraturas patológicas (33,3%), cirurgias extensas (61,1%) e amputações (27,8%). Nessas regiões, o tempo pré-cirúrgico foi mais longo (médias de 36 e 39 dias) comparado ao Sul e Sudeste (27 e 33 dias, respectivamente).

**Conclusões:**

Os resultados refletem disparidades regionais no Brasil, sugerindo que condições socioeconômicas influenciam os desfechos clínicos. Estes achados são importantes para melhorar o cuidado oncológico ortopédico em regiões desfavorecidas do país. ***Nível de Evidência III; Coorte Retrospectiva.***

## BACKGROUND

Giant cell tumor of bone (GCTB) is a benign primary bone tumor that is known to be locally aggressive. Histologically, GCTB is characterized by the presence of numerous multinucleated giant cells surrounded by a monotonous population of mononuclear stromal cells. This tumor predominantly affects adults between the ages of 20 and 40, with a predilection for the epiphyses of long bones. There is no significant gender disparity, although it appears to be more common in females. The incidence of GCTB is not accurately known, although registries from Japan, Australia, and Sweden have estimated it at 1.03 to 1.33 cases per million per year.^1–3^

The standard treatment for any primary bone tumor is complete surgical resection; however, in selected cases, treatment with the receptor activator of nuclear factor kappa-B ligand (RANK-L) inhibitor denosumab, bisphosphonate therapy, or even radiation therapy may be employed. Delayed treatment and local recurrence are issues of great importance, as they may lead to impairment of joint function, bone loss, and a theoretical high risk of metastasis. Despite its local aggressiveness and considerable risk of local recurrence (10–75%), GCTB has a favorable prognosis in terms of overall survival (approximately 98% at 5 years). Due to their rarity, primary bone tumors should be managed at referral centers, as diagnosis and treatment are challenging and expensive.^4,5^

In Brazil, 70% of the population is served by the public Unified Health System (SUS), established in 1988. The system has long been overburdened, leading to lengthy waiting times for specialized treatment. Furthermore, there are substantial variations in care quality among the country’s geopolitical regions due to the variation in investments made by each state of the federation. Patients with bone tumors rely on the availability of appointments with specialists in their respective city or state. In many cases, delaying treatment can result in clinical deterioration and poor outcomes.^6^

Despite the large number of patients with GCTB treated at referral centers in Brazil, few studies on this topic have been published to date, and a gap in information regarding the epidemiological profile of this tumor persists. This study aims to elucidate patient and tumor characteristics, management practices, and outcomes in the unique context of Brazil – a middle-income country with a publicly funded, universal health system serving a very large population across different geopolitical regions. Our findings could help redirect financial and human resources to optimize outcomes.^3,7,8^

## METHODS

This research project (number 94280918.0.0000.5327) was approved by the ethics committee of the coordinating center (Hospital de Clínicas de Porto Alegre, state of Rio Grande do Sul) and, subsequently, by the 18 participating centers. Orthopedic follow-up was performed according to the routine protocol of each center. Case report forms were completed and sent to the coordinating center. The inclusion criteria for this study were: histopathological diagnosis of GCTB; treatment of the primary tumor at the same health care facility in which it was diagnosed; and availability of complete medical records. Patients of all ages with tumors in the appendicular and axial skeleton were included. The lead researcher at each participating center reviewed the data from medical records and then sent them via e-mail to the coordinating center. All lead researchers were active members of the Brazilian Association of Orthopedic Oncology (ABOO) while the study was ongoing.

General variables such as age, sex, and region of the country where the patient was treated were extracted from medical records, as were specific characteristics related to the tumor, such as anatomical location, presence of metastases, type of surgery, use of cavity filling material, adjuvant treatment, Campanacci radiological classification, presence of pathological fracture, local recurrence, death, and use of denosumab. For analysis, all variables were stratified by the geopolitical region of Brazil (North, Northeast, South, and Southeast) in which the patient was treated. Cases from 1989 to 2021 were included. The sample consisted of 653 patients. Of these, we excluded 10 due to lack of data on initial treatment and one confirmed diagnosis of tenosynovial giant cell tumor. Therefore, we retrospectively analyzed the records of 643 patients with a histologically confirmed diagnosis of GCTB treated at 16 health care facilities across 8 Brazilian states. Two centers which approved the study protocol ultimately did not include patients. The North, Northeast, South, and Southeast regions included 18, 87, 170, and 368 patients, respectively.

Quantitative variables were expressed as mean (SD), while categorical variables were expressed as absolute and relative frequencies. Data were analyzed in SPSS version 21.0.

## RESULTS

The general characteristics of the sample are shown in Table 1. The cohort comprised 351 (54.6%) women and 292 (45.4%) men, with a mean age of 32 (SD, 13) years, ranging from 8 to 77 years. The median follow-up was 7.1 years. Campanacci stage I and II tumors accounted for 38.6% (n=248) of cases, and Campanacci stage III tumors, for 61.4% (n=395). Data were missing in 3 cases. Appendicular tumors accounted for 92.1% of cases (n=597), the knee being the most frequently affected level (52.2%; 175 distal femur, 133 proximal tibia, and 27 proximal fibula), followed by hand and wrist (18.7%; 74 distal radius, 26 hand, and 20 distal ulna), foot and ankle (9.6%; 29 distal tibia, 18 in metatarsus, talus, and calcaneus, and 1 distal fibula) and scapular girdle (6.0%; 30 proximal humerus, 6 scapula, and 2 clavicle). Tumors located in the axial skeleton accounted for 7.1% of cases (n=46), with the most common sites being the pelvic girdle (n=20), sacrum (n=12), and spine (n=12).

**Table 1:** General characteristics.

Variables	n (%)
Campanacci grade	
I/II	248 (38.6)
III	395 (61.4)
Pulmonary metastasis	
No	616 (94.9)
Yes	33 (5.1)
Pathological fracture	
No	551 (85.7)
Yes	92 (14.3)
Type of surgery	
Curettage	323 (50.2)
Marginal/wide	278 (43.2)
Amputation	24 (3.7)
Not performed	17 (2.6)
Type of filling (n=323)	
Cement	271 (84)
Cement and bone graft	11 (3.4)
Bone graft alone	23 (7.1)
No filing	14 (4.3)
Missing	4 (1.2)
Adjuvant (n=323)	
None	50 (15.4)
One	131 (40.5)
Combined (two or more)	142 (44.0)
Local recurrence*	114 (18.2)
Denosumab*	
No	542 (86.6)
Yes	84 (13.4)

* Only surgically treated patients (n=626).

**Figure 1:**
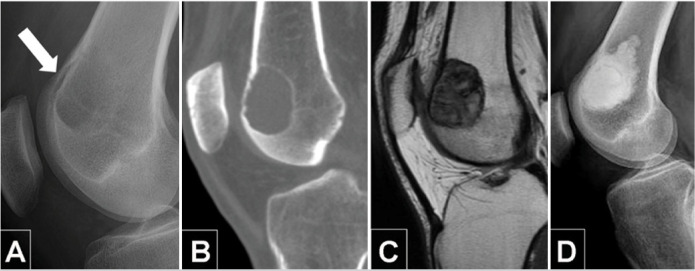
A) Preoperative radiograph and B) computed tomography scans displaying a meta-epiphyseal lesion causing endosteal erosion (Campanacci II) and expanding the anterior cortex of the distal femur (arrow). C) Contrast-enhanced MRI of the knee revealing the medullary and cortical boundaries of a giant cell tumor of bone (GCTB). D) Postoperative radiograph depicting the cavity filled with cement after curettage. Despite endosteal involvement and medullary osteolysis, joint structure and function were successfully restored.

**Figure 2:**
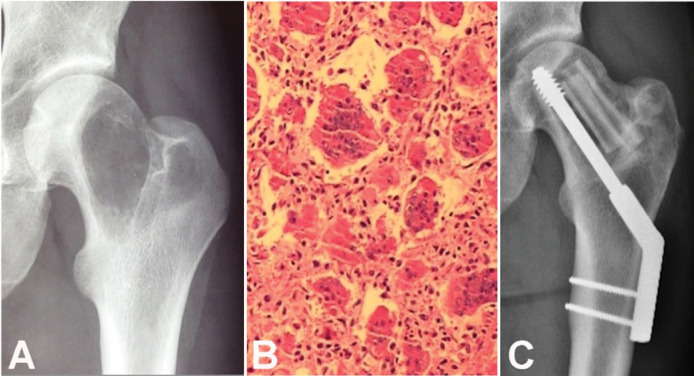
A) Preoperative radiograph showing an osteolytic lesion partially confined to the left proximal femoral epiphysis (Campanacci II). B) Microscopic view of the bone lesion after a core needle biopsy, revealing multiple multinucleated giant cells within a stroma of mononuclear cells. C) 12-month postoperative follow-up radiograph after curettage, fulguration, and alcoholization of the tumor bed, plate fixation, and filling with xenograft and autologous fibula. There was complete incorporation of the graft.

Surgery was performed in 626 patients, with curettage in 50.2% (n=323), marginal or wide resection in 43.4% (n=279), and amputation in 3.7% (n=24). Patients treated without surgery accounted for 2.6% (n=17) of cases. The reasons for not operating were unresectable tumors, major surgical morbidity, personal and family decisions, and poor clinical condition. Of the 323 patients who underwent curettage, 15.4% (n=50) were treated without adjuvant therapy, 40.5% (n=131) received only one adjuvant (ethanol or fulguration or drilling), and 44.0% (n=142) received two or more combined adjuvants. The bone defect was filled in 95.7% (n=309) of cases, and the remaining 4.3% (n=14) received no filling. Bone cement (PMMA) was used in 271 cases (Figure 1), cement combined with bone graft in 10 cases, and bone graft alone in 23 cases. (Figure 2) There were no data on the specific type of reconstruction after wide and marginal resections.

A total of 97 patients were treated with denosumab. Indications for use were preoperative cytoreduction (57 cases), local recurrence (14 cases), tumors associated with major surgical morbidity (5 cases), pulmonary metastases (4 cases), and other reasons (4 cases). Patients treated with preoperative denosumab showed a local recurrence rate of 14% (8/57). Denosumab was used in only 15% (97/643) of the patients due to the scarcity of resources at public health facilities.

**Figure 3:**
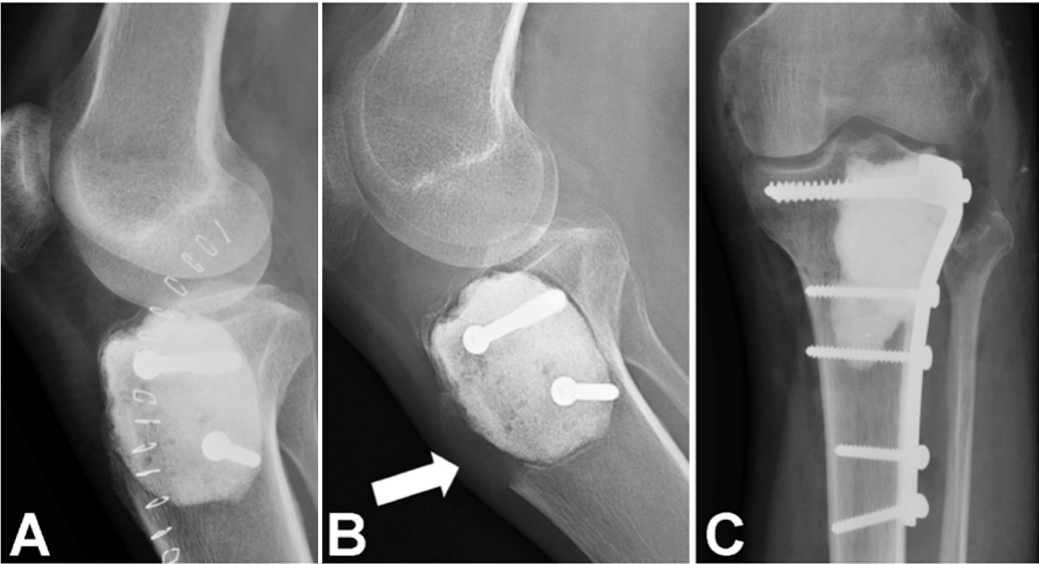
A) Immediate postoperative radiograph of the knee after curettage of a proximal tibial GCTB; cement is visible filling the defect. B) After 18 months, there was local recurrence with severe osteolysis adjacent to the cement and rupture of the cortical bone. C) Anteroposterior radiograph showing surgical treatment of the recurrence (curettage, drilling, fulguration, and alcoholization with 99% ethanol) with successful joint preservation. Bone cement and reinforcement with a proximal tibia plate were used.

Patients diagnosed with GCTB had pulmonary metastases present at initial staging in 5.1% of cases (n=33), and pathological fractures in 14.3% (n=92). Among those with metastatic GCTB, the mortality rate was 15.1% (5/33). The rate of local recurrence among those who underwent surgery was 18.2% (n=114). As expected, curettage had a higher rate of local recurrence (24.4%) compared to wide and marginal resections (12.5%). (Table 2) The local recurrence rate according to affected bone was 30% (4/12) in the sacrum, 26.6% (8/30) in the proximal humerus, 25.6% (19/74) in the distal radius, 17.1% (30/175) in the distal femur, 14.2% (19/133) in the proximal tibia (Figure 3), and 10% (2/20) in the pelvis.

**Table 2:** Local recurrence rate and cohort characteristics.*

Variables	Local recurrence n=114 (%)
Sex – n(%)	
Female	71 (11%)
Male	43 (7%)
Campanacci grade – n(%)	
I/II	40 (6%)
III	74 (12%)
Anatomical site – n(%)	
Sacrum and spine	6/22 (27%)
Humerus and scapula	10/37 (27%)
Wrist and hand	25/120 (21%)
Foot and ankle	13/61 (21%)
Pelvis and hip	4/31 (13%)
Knee	53/333 (16%)
Elbow	2/12 (16%)
Other sites	1/10 (10%)
Skeletal distribution – n(%)	
Axial	10/53 (19%)
Appendicular	104/573 (18%)
Type of surgery – n(%)	
Curettage	79/323 (24%)
Marginal/wide	35/279 (12%)
Amputation	0 (0%)
Type of filling – n(%)	
Cement (PMMA)	60/271 (22%)
Cement and bone graft	1/10 (10%)
Bone graft alone	10/23 (43%)
No filling	6/14 (42%)
Adjuvant – n(%)	
None	18/50 (36%)
One	33/131 (25%)
Combined (two or more)	28/142 (20%)
Preoperative denosumab	8/57 (14%)

* Only surgically treated patients (n=626).

The analysis stratified by geopolitical region demonstrated higher rates of lung metastases (12.6%) and Campanacci stage III tumors (88.9%) in the Northeast and North, respectively. Likewise, the North region had higher rates of pathological fractures (33.3%), wide resection (61.1%), and amputations (27.8%). Finally, there was a longer interval between tumor diagnosis and primary surgery in the Northeast and North regions (36 and 39 days) compared to the South and Southeast regions (27 and 33 days). (Table 3)

**Table 3:** Sample characteristics stratified by Brazilian geopolitical region.

Variables	South	Northeast	Southeast	North
	(n=170; 26.4%)	(n=87; 13.5%)	(n=368; 57.2%)	(n=18; 2.8%)
Campanacci grade – n(%)				
I/II	62 (36.5)	27 (31.0)	157 (42.7)	2 (11.1)
III	108 (63.5)	60 (69.0)	211 (57.3)	16 (88.9)
Pulmonary metastasis – n(%)	5 (3.0)	11 (12.6)	16 (4.6)	1 (5.6)
Pathological fracture – n(%)	27 (15.9)	9 (10.3)	50 (16.6)	6 (33.3)
Type of surgery – n(%)				
Curettage	99 (59.3)	51 (58.6)	171 (48.3)	2 (11.1)
Marginal/wide	65 (38.9)	30 (34.5)	173 (48.9)	11 (61.1)
Amputation	3 (1.8)	6 (6.9)	10 (2.8)	5 (27.8)
Local recurrence – n(%)	35 (21.0)	16 (18.4)	62 (17.5)	1 (5.6)
Time between diagnosis and surgery (days)**	27 (6-69)	36 (18-78)	33 (6-80)	39 (20-91)

** Median (interquartile range).

## DISCUSSION

In Brazil, several referral centers for the treatment of bone tumors have been accumulating cases of GCTB in their records for decades. Despite this large number of patients, only a few case series have been published. To address this issue, we developed a research project which would collate patients from the country’s main referral centers and create a multicenter database. This first Brazilian epidemiological study on GCTB included 643 patients and gave further support to some findings already described in the international literature. We consider that the local recurrence rate was high (18.2%), although previous cases series have reported recurrence rates ranging from 10% to 75%.^5,9,10^ The high recurrence rate observed in the curettage cohort (24.4%) can be partially attributed to the inclusion of patients treated since the late 1980s, when the range of adjuvant therapies, diagnostic imaging techniques, and treatment modalities available was limited. Even though curettage entailed a twofold local recurrence rate relative to resection, many authors still prefer this technique in order to protect the bone structure and ensure joint function.^9–11^ Moreover, due to the large proportion of patients with Campanacci stage III tumors (61.4%), we hypothesize that diagnostic and treatment delays may have occurred in some of the cases. This may also reflect the difficulty of accessing specialized care through the public health system.

Tumors located in the axial skeleton have been observed to have higher rates of local recurrence compared to those in the appendicular skeleton. Balke et al.^12^ examined nineteen patients with GCTB of the spine and sacrum and reported an overall local recurrence rate of 66.7%, and 15.4%, respectively. Likewise, Junming et al.^13^ evaluated 22 patients with cervical spine GCTBs treated consecutively; the local recurrence rate was 71.4% for subtotal resection, and 7.7% for total spondylectomy.^12–14^ Generally, this outcome has been linked to the difficulty of surgical approaches to the spine and pelvis, incomplete resections, larger tumors, and the biological characteristics of the neoplasm.^14–16^ In our cohort, we observed a higher rate of local recurrence for lesions located in the sacrum (33%, 4/12) and spine (20%, 2/10) than for most appendicular lesions.

Additionally, we observed a high rate of local recurrence in our patients with tumors of the proximal humerus and distal radius.^17,18^ In the proximal humerus, a local recurrence rate of 26.6% was observed, while curettage and resection of distal radius lesions was associated with local recurrence rates of 35% and 16%, respectively. Theoretically, this higher risk of local recurrence may be related to the high percentage of stage III tumors at diagnosis, proximity to blood vessels, incomplete curettage, and the biological behavior of the tumors.^5,10,18^ The prevalence of lung metastasis is increased in patients with local recurrence, and considerably worsens survival rates.^14,19^ In our study, the metastasis rate was indeed higher (13%) in patients with local recurrence, and the rate of death in metastatic patients was 15%. These data corroborate the findings of previous studies.

Denosumab has become a leading pharmacological option for the treatment of GCTB. Indications for use include locally extensive disease, unresectable tumors, major surgical morbidity, pulmonary metastases, local recurrence, preoperative or postoperative treatment, and even pain control.^20^ In general, tumors with poor prognosis are treated with denosumab. Examples include tumors located in the pelvis, spine, or even in the long bones when overly aggressive. As a result, poorer outcomes in terms of local recurrence, metastases, and death seemingly associated with the use of denosumab may be explained by patient selection bias. Moreover, treatment with curettage after denosumab may be surgically challenging due to extensive bone formation inside the lesion. Theoretically, neoplastic cells confined to the trabecular bone that has not been removed would be the reason for local recurrence. In our series, preoperative denosumab followed by curettage did not increase the rate of local recurrence.

GCTB is a highly curable neoplasm; however, several factors can interfere with the success of treatment. Studies have demonstrated that delayed diagnosis and treatment of GCTB correlate with larger tumors, higher recurrence rates, and higher rates of local complications. Furthermore, patients in whom diagnosis or treatment of GCTB are delayed are more likely to require more aggressive treatments, such as amputation or chemotherapy. Additionally, we identified that the disparities in development and health investments among Brazilian geopolitical regions were reflected in the characteristics and outcomes of our patients with GCTB.^6^ Despite the limited scope of our cohort, regions with a lower human development index and annual per capita income, such as the North and Northeast, showed higher rates of pulmonary metastases, stage III tumors, pathological fractures, wide resections, and amputations, as well as a longer time between diagnosis and primary treatment, as compared to states in the more highly developed South and Southeast regions. Measures to promote and protect health should be taken in order to reduce these inequalities between regions in Brazil. It should be borne in mind that treatment of GCTB is the responsibility of high-complexity referral centers, and investments must be made as needed in order for individuals to have access to these institutions as quickly as possible. The findings in this cohort are certainly replicable for other musculoskeletal tumors, particularly regarding a similar situation of delayed diagnosis, but with even worse prognosis. The main limitation of this study which may have had an effect on our findings is that the sample comprised patients who were treated in different decades, by surgeons with varying levels of experience in the treatment of GCTB, and with great variation in the availability of imaging modalities, surgical materials, and histopathological analysis. Nonetheless, to the best of our knowledge, the 640 patients included in this study comprise the largest case series with epidemiological data on GCTB in Latin America.

## CONCLUSIONS

Among patients with GCTB in Brazil, characteristics such as sex, age, tumor aggressiveness, anatomical location, type of surgery performed, local recurrence rate, and metastases were similar to those described in the international literature. Patients treated in geo-political regions with a lower HDI and per capita income presented higher rates of pathological fractures, metastases, larger tumors, and amputations, as well as longer delays between diagnosis and treatment. Despite the large cohort size, limitations and possible biases of this study should be considered.
